# 

**Published:** 1883-12

**Authors:** 


DECEMBER, 1383.

THIRTIETH YEAR.

### HALL'S JOURNAL OF HEALTH

Guaranteed Circulation 200,000 Copies in 12 Months.

*E. H. GIBBS, A.M., M.D., Editor,*

No. 21 CLINTON PLACE, EIGHTH STREET, NEW YORK.

TERMS: $1 a Year. Single number, 10 cts.

